# Pluripotent stem cell-based therapies and their path to the clinic

**DOI:** 10.1016/j.stemcr.2023.06.010

**Published:** 2023-08-08

**Authors:** Kazuo Takayama, Shinya Yamanaka

**Affiliations:** 1 Center for iPS Cell Research and Application (CiRA), Kyoto University, Kyoto, Japan; 2 CiRA Foundation, Kyoto, Japan; 3 Gladstone Institute of Cardiovascular Disease, San Francisco, USA

**Figure:**
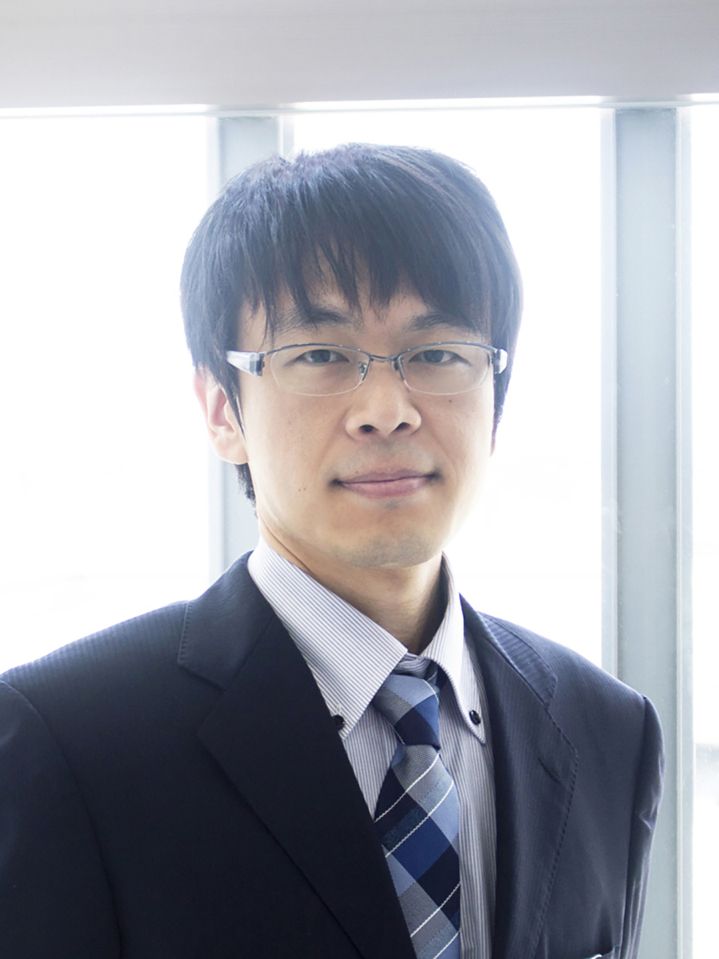

**Figure:**
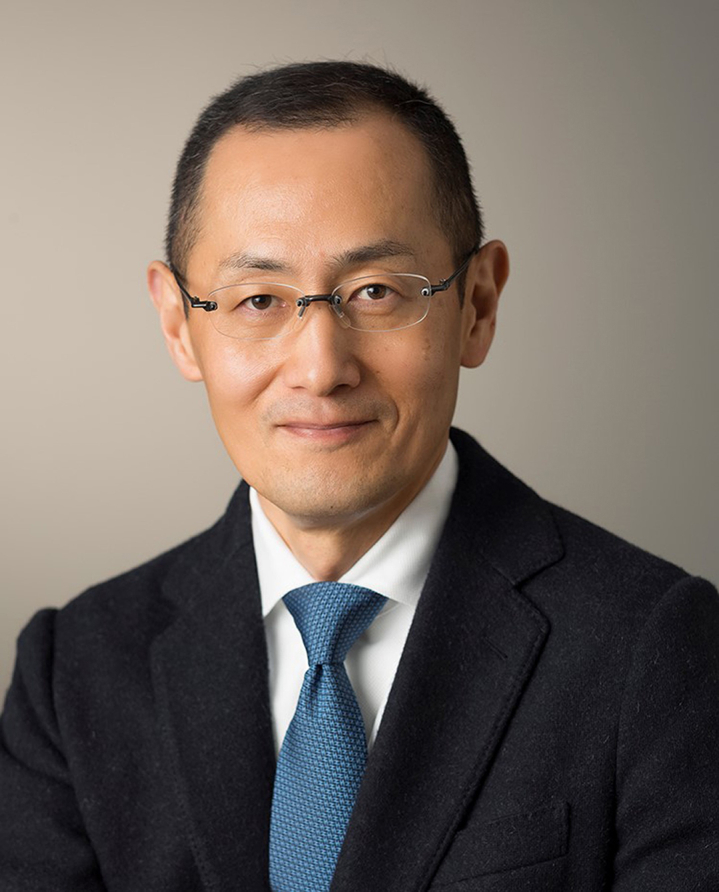

Welcome to this special issue, focusing on the potential of pluripotent stem cell (PSC)-based therapies and their paths toward clinical application. Since the establishment of human embryonic stem (ES) and induced pluripotent stem (iPS) cells in 1998 and 2007, respectively, significant progress has been made in differentiating PSCs into a broad range of somatic cells. We are now closer than ever before to having highly functional PSC-derived somatic cells at purity for transplantation therapies to complement damaged or diseased organs and restore their physiologic functions. Like organ transplantation, PSC-based therapies have the potential to regenerate damaged organs that cannot otherwise be healed by using small-molecule or antibody-based drugs.

In this issue, Kobold et al. present an overview of the history and current status of clinical studies utilizing human PSCs. Since the early 2010s, many clinical studies employing human ES cells have been initiated. By 2018, the number of such studies using human iPS cells had skyrocketed. Many PSC-based therapies are currently being tested to treat various pathologic conditions, including different neoplasms and diseases of the eye, adnexa, and circulatory system. However, there are still many diseases that require further efforts to interrogate the true potential of PSC-based therapies. To advance the use of PSC-based therapy to treat a wider range of pathologic conditions in the future, we must continue with extensive basic and clinical research to establish both efficacy and safety for such new therapies.

Although clinical research on PSC-based therapy for liver diseases has not received as much attention, there is much hope for it to become a real alternative to living-donor liver transplantation. Cardinale et al. provided a comprehensive summary of the recent studies on cell-based therapy for liver diseases. In addition, artificial livers generated through bioengineering efforts are now considered to be a viable option. Aside from traditional cell or organ transplantation to restore impaired liver function, transplantation aimed at treating the microenvironment, such as inflammation, in the liver is also an effective therapeutic strategy. Concurrent research efforts in both basic and clinical studies will be crucial in making PSC-based therapy for liver diseases a reality.

This special issue includes three research papers reporting basic research on the development of PSC-based therapy. (1) Martinez-Curiel et al. demonstrated the *in vitro* and *in vivo* production of myelinating oligodendrocytes from a human iPSC-derived long-term neuroepithelial stem cell (NES) line, which also gives rise to neurons with the capacity to integrate into stroke-injured, adult rat cortical networks. This long-term NES cell line holds promise for repairing both damaged neural circuits and demyelinated axons. Such a strategy has potential as a new treatment for neurological disorders and injuries, such as ischemic stroke. (2) To successfully carry out PSC-based therapy, it is of utmost importance to ensure not only its efficacy but also its safety. The exclusion of unwanted cells, such as undifferentiated cells, is vital to ensuring the safety of PSC-derived somatic cells for transplantation. Taga et al. report a method for purifying adrenocorticotropic hormone-secreting PSC-derived pituitary cells based on epithelial cell adhesion molecule, a cell-surface marker for pituitary cells. In the study, they successfully purified pituitary cells and kept off-target cells to a minimum. The transplantation of purified pituitary cells offers an effective and safe solution to treating hypopituitarism. (3) To maximize the therapeutic efficacy of PSC-based therapy, combining it with gene therapy is one of the major focuses in the development of so-called next-generation cell therapy. Transplanting cells transduced with specific genes to modify their functions or properties could be an effective means to enhance their therapeutic effects. Laperle et al. demonstrated the effectiveness of glial cell-derived neurotrophic factor (GDNF)-transduced iPS cell-derived neural progenitor cells (iNPC-GDNFs) as a novel approach to treating neurodegenerative diseases. The transplantation of iNPC-GDNFs into the subretinal space of a Royal College of Surgeons rodent model of retinal degeneration was shown to preserve photoreceptors and visual functions. Prolonged motor neuron protection was also observed in an SOD1G93A amyotrophic lateral sclerosis (ALS) rat model upon receiving iNPC-GDNF transplants in the spinal cord. As such, the authors illustrated the potential of using such PSC-derived cells to impart significant neuroprotection in these debilitating models of retinal degeneration and ALS. By using gene transfer technology to improve or even generate new functionalities of somatic cells derived from PSCs, it is up to the imagination of stem cell scientists to find creative new ways to enhance the therapeutic potential of PSC-based implants.

As basic research on PSC-based therapy continues to forge ahead, clinical research is also striving forward synchronously at a rapid pace. Nonetheless, the necessity for stringent rules to evaluate the safety and efficacy of novel therapies remains. Hirai et al. recently highlighted how cell-based therapeutic products (CTPs) should not be assessed using the same criteria as those for small molecule drugs and antibodies, thus necessitating the creation of new evaluation methods. As PSC-based therapies often employ emerging technologies, such as gene therapy, it is essential to update our evaluation methods and standard guidelines continuously to accompany new technological advances. Furthermore, it will be vital to establish international standardization for the testing and implementation of PSC-based therapies that can be applied universally across all countries.

In order to properly evaluate PSC-based therapies during clinical trials, various challenges—such as determining the efficacy and safety of CTPs—must be addressed. The ISSCR Clinical Translation Committee outlined key considerations that the scientific and medical communities must contemplate to advance PSC-based therapies forward: (1) choosing the appropriate stem cell line and meticulously assessing the genomes of both the starting and final product; (2) acclimatizing to GMP manufacturing, reagent validation, and supply chain management; (3) enduring product delivery issues and additional regulatory challenges; (4) understanding the relationship between clinical trial design and preclinical studies; and (5) realizing market approval requirements, pathways, and partnerships needed.

Expectations surrounding PSC-based therapy are higher than ever as it holds promise to overcome a wide range of intractable diseases. However, such heightened anticipation has also led to issues related to unproven stem cell usage. As described by Dulak and Pecyna and Kawam et al., unproven stem cell interventions necessitate vigilance from healthcare professionals, researchers, and patients to avert their use. It is essential to impart a better understanding of PSC-based therapy, including its advantages and drawbacks, how it fares against other available options, and its financial costs. For individuals to readily access and comprehend this information, all stakeholder groups must consistently convey clear and concise information.

At present, the market size for PSC-based therapy is smaller than that for small molecule, protein-based, and antibody-based drugs. Nevertheless, we are confident that with the emergence of innovative PSC-based therapy, the market size will rapidly expand, analogous to how RNA-based medicine boomed in the past few years. For PSC-based therapy to become more widespread and readily available, we must overcome many obstacles. It is unimaginable that it has only been 25 and 16 years since human ES and iPS cells were established, respectively. Finally, we have arrived at the stage of testing these new therapeutic modalities in clinical trials. Our mission is to continue to persistently run so that we can create a future in which life-threatening illnesses are no longer life threatening because PSC-based therapies will be available to treat any pathologic conditions where traditional approaches have failed.

